# The Relationship Between Health Expenditure, CO_2_ Emissions, and Economic Growth in G7: Evidence from Heterogeneous Panel Data

**DOI:** 10.1007/s13132-023-01349-y

**Published:** 2023-04-04

**Authors:** Melina Dritsaki, Chaido Dritsaki

**Affiliations:** 1 Department of Economics & Laboratory of Applied Economics, University of Western Macedonia, 52100 Kastoria, Greece; 2 Department of Accounting and Finance, University of Western Macedonia, Kila Kozanis, 50100 Kozani, Greece

**Keywords:** Health expenditures, CO_2_ emissions, Economic growth, Panel data, Group of Seven (G7), C22, C23, I10

## Abstract

Τhe current paper examines the relationship between per capita health care expenditures, per capita CO_2_ emissions, and per capita gross domestic product (GDP) in G7 countries. At the beginning, we examine the cross-sectional dependence and the slope homogeneity between the countries. Then, the second-generation unit root test is applied using the Pesaran, CIPS (2007) test, while for the cointegration test, the Westerlund (Oxford Bulletin of Economics and Statistics 69(6):709-748, 2007) test was applied. The long -run panel cointegration coefficients were analyzed with the augmented mean group (AMG) estimators, which allow the cross-sectional dependence and heterogeneity. Finally, the test by Dumitrescu and Hurlin (Economic Modelling 29(4):1450-1460, 2012) was used in order to check for causality taking into account the heterogeneity and cross-sectional dependence on panel data. The preliminary analyses show that variables are cross-sectional-dependant and heterogenous and are first-order stationary. Cointegration test by Westerlund (Oxford Bulletin of Economics and Statistics 69(6):709-748, 2007) which allows heterogeneity and cross-sectional dependence show that there is a stable and long-run relationship between variables. Moreover, the long-run coefficients which were estimated with the AMG approach are found to be statistically significant and positive for the GDP per capita, and negative in the case of greenhouse gas emissions per capita. Finally, causality test by Dumitrescu and Hurlin (Economic Modelling 29(4):1450-1460, 2012) revealed a unilateral causality from greenhouse gas emissions per capita towards health expenditure per capita for all G7 countries.

## Introduction

Climate change has become an undeniable fact which represents a severe threat for the sustainable development of human survival, society, economy, and environment. There is a significant relation between high greenhouse gas emissions and climate change on public health. The degradation of the quality of the environment worldwide has a significant impact on what we refer to as “healthy living.” The impact of environmental factors is measured through the greenhouse gas emissions on health care government spending, knowing that pollution has detrimental effects on people’s health. As the environmental quality is becoming worse, the downgrade of global environmental quality has become a serious challenge for healthy living. The particles from the burning of fossil fuels, methane, nitrous oxide, sulphur dioxide, and CO_2_ emissions significantly contribute to the global climate change which became a topic under investigations from various research sectors.

For the G7 team, climate policy has become the main priority in order to reduce C0_2_ emissions. Compared to 10.1 GtCO2 emissions in 1999, G7 was responsible for 8.6 GtCO2 in 2019, a reduction of approximately 1.5 GtCO2 (15.3%). These results suggest that most of these reductions, approximately 1.3 GtCO2 (12%) were due to climate change and not due to wider socioeconomic changes. The greater part of this outcome begun after 2009, when significant laws were imposed, such as the UK law on climate change.

Table 1 shows the CO_2_ emissions volatility between 1999 and 2019 related to the relative impact of climate legislation “impact of laws” versus other changes without legislation.

**Table 1:** CO_2_ emissions volatility between 1999 and 2019 in G7 countries

G7 countries	CO_2_ in 1999 (Mt CO2)	Change in CO_2_ without laws (1999 to 2019, %)	Policy induced change (1999 to 2019, %)	Carbon emissions in 2019 (in % of 1999)
Canada	551	7.3%	−9.5%	97.8%
France	424	2.4%	−18.7%	83.7%
Germany	907	13.9%	−23.4%	90.5%
Italy	469	−10.6%	−17.1%	72.3%
Japan	1240	10.3%	−15.1%	95.2%
UK	569	−25.4%	−16.1%	58.5%
United States	5,950	−6.7%	−9.2%	84.0%

Source: Own calculations, based on Eskander and Fankhauser.

The results from the table above contradict the notion that wider socioeconomic changes and market forces have reduced emissions, for reasons which have little to do with politics around climate. In June 2021, G7 hosted the annual word summit in Cornwall, Southwest England. The issues discussed were a response to COVID-19 pandemic and climate change. The British prime minister asked G7 to work on a global approach towards pandemics and to secure an equal distribution of COVID-19 vaccinations so that future pandemics will be prevented. The official G7 website states that “a greener, more prosperous future” was a priority in the meeting.

**Fig. 1:**
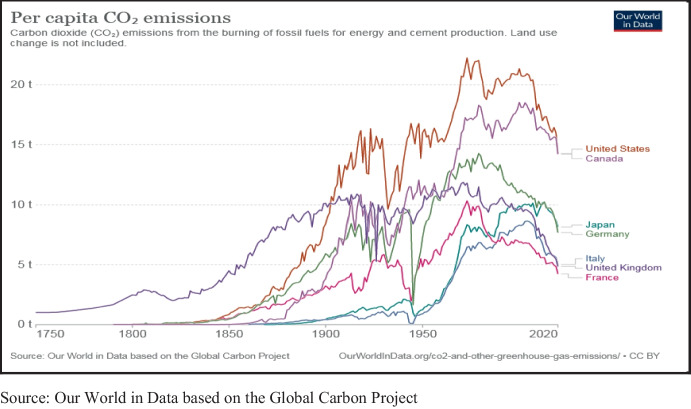
Pathway of the per capita CO_2_ emissions of G7 from 1750 to 2020

**Figure 2:**
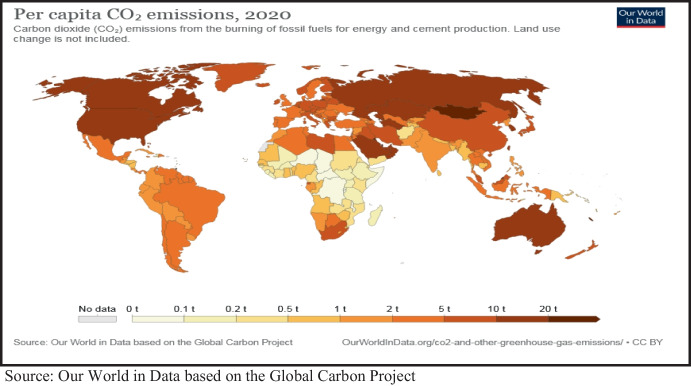
Per capita CO_2_ emissions globally in 2020

Figure 1 shows the CO_2_ emission pathway from the burning of fossil fuels for energy and cement production in G7 from 1750 to 2020 (Land use change is not included). Figure 2 presents the per capita CO_2_ emissions for 2020 globally.

From Fig. 1, we see there is a significant reduction of the per capita CO_2_ emissions in all G7 countries from 2002, after the Kyoto Protocol validation, although the reduction in the UK, Germany, and France occurred a few years later.

The picture above shows the per capita CO_2_ emissions for 2020 globally. The dark red represents the countries with the highest per capita CO_2_ emissions (Qatar, Mongolia, Kuwait, Brunei, Bahrain), ranging from 20 to 40 tons. In red, the countries with 10-20 tons per capita (Saudi Arabia, Australia, the USA, Canada, Russia). In white, we can see the countries which have 0–1 tons per capita CO_2_ emissions and are primarily African countries (Democratic Republic of Congo, Central African Republic, Burundi, Chad, Ethiopia, Burkina Faso, Eritrea, Gambia, Cameroon, Comoros, Kenya, Cote d’Ivoire, Ghana, Congo, Angola, Cambodia, Cape Verde, etc.).

During the last decades, however, the relations between economic growth and the degradation of the environment with healthcare expenditure have received a growing attention in the literature review. Nevertheless, the negative external effects of low environmental quality due to the impact on health have been neglected. Several studies confirm that GDP serves as one of the main impact factors which affected volatilities in healthcare expenditure among countries.

Although many papers have been written about the health expenditure sector and many analyses have been developed for the factors affecting health expenditure (Akca et al., 2017, Amiri et al., 2021, Omri et al., 2022), the current paper deals with a very contemporary subject. It deals with the impact of CO_2_ emissions (greenhouse gas emissions) on the expenditure of healthcare especially on G7 countries, which has been investigated by very few empirical studies so far. More specifically, this paper mainly uses the data of per capita healthcare expenditure, per capita GDP as an index of economic growth and per capita greenhouse gas emissions (corresponding on CO_2_ emissions) from 2000 to 2018 derived from World Bank database. The long-run panel cointegration coefficients are analyzed with augmented mean group (AMG) estimators, which allow the cross-sectional dependence and heterogeneity. Granger causality test examines the short-run causality for each country and the test of Dumitrescu and Hurlin (2012) is used for detecting the causality of G7 countries, taking into account the heterogeneity and cross-sectional panel data.

It is obvious that studies that combine time series and cross-sectional data for the estimation of relationship between per capita healthcare expenditure, per capita carbon dioxide emissions and per capita gross domestic product are missing from the existing literature. For example, the recent paper by Ageli (2022) uses Bootstrap Autoregressive Distributed Lag (BARDL) cointegration model to examine if there exists a short- and long-run relationship between per capita health expenditure, per capita GDP, and per capita CO_2_ emissions in Saudi Arabia for the period 1995–2021.

### Research Questions and Hypotheses

The research questions and hypotheses that will be examined and are related to the correlation of per capita healthcare expenditure, per capita greenhouse gas emissions, and per capita gross domestic product on G7 countries are the following:

- RQ1: Which is the relationship between *GDP per capita and Health expenditure per capita* on G7 countries;
- H01: There is *no statistically significant* relationship between GDP per capita and Health expenditure per capita.
- Ha1: There is *statistically significant* relationship between GDP per capita and Health expenditure per capita.
- RQ2: Which is the relationship between *greenhouse gas emissions per capita and Health expenditure per capita* on G7 countries;
- H01: There is *no statistically significant* relationship between greenhouse gas emissions per capita and Health expenditure per capita.
- Ha1: There is *statistically significant* relationship between greenhouse gas emissions per capita and Health expenditure per capita.
- RQ3: There is a *short-run causal relationship between GDP per capita and Health expenditure per capita* on G7 countries
- H01: There is *no short-run causal relationship* between greenhouse gas emissions per capita and Health expenditure per capita.
- Ha1: There is *a short-run causal relationship* between greenhouse gas emissions per capita and Health expenditure per capita.
- RQ4: There is a *short-run causal relationship between greenhouse gas emissions per capita and Health expenditure per capita* on G7 countries
- H01: There is *no short-run causal* relationship between greenhouse gas emissions per capita and Health expenditure per capita.
- Ha1: There is a *short-run causal* relationship between greenhouse gas emissions per capita and health expenditure per capita.

The aims of the current study which contribute to the empirical literature review are the following:

First, as mentioned before, there is a limited number of studies which have analyzed the impact of environment and GDP on healthcare expenditure in the case of G7 countries.

Second, the study represents the environment with the per capita CO_2_ emissions.

Third, the investigation of the impact of determinants of per capita healthcare expenditure has been achieved through panel cointegration analysis of Westerlund (2007) as well as causality analysis of Dumitrescu and Hurlin (2012) using STATA 14.0 and EViews 12.0.

The remainder of this paper organized as follows: the “Literature Review” section presents a literature review in relation to the topic under investigation. The “Data” section presents the data and the “Methodology” section the methodology. The “Empirical Analysis” section explains the main results of the study, and the “Discussion” section presents a discussion. Finally, the “Conclusions” section presents the conclusions of the study.

## Literature Review

The quality of the environment possesses a crucial role in the process of economic growth. The deterioration of environmental quality can be explained from the increase of CO_2_ emissions and is due to the use of energy resources such as oil, natural gas, and carbon. It is estimated that almost each year seven million people die from diseases related with CO_2_ emissions. The increase of per capita CO_2_ emissions in China, Turkey, Asia, Brazil, Iran, and India has caught the attention of many researchers to investigate the relations between CO_2_ emissions and health care expenditures with development in these countries.

The structure of the literature review is based on recent studies that examine three vital relationships directing the empirical questions addressed on this paper. This section starts with a review of the relationship between healthcare expenditure and economic growth. Afterwards, a review on the relationship between healthcare and CO_2_ emissions is presented, and finally, an empirical research examining the impact of CO_2_ emissions and economic growth is reviewed.

### Health Expenditure and Economic Growth

Many studies have ascertained that there is a direct effect between economic growth and healthcare expenditure. Researchers have used various econometric methodologies to analyze the relationship οn different countries and concluded that there exists a positive correlation between these two variables.

Some of the most recent papers are referred below:

Gok et al. (2018) study the relation between healthcare expenditure and economic growth in emerging BRICS countries as well as Mexico, Indonesia, South Korea, and Turkey from 2008 to 2012. Healthcare expenditure was used as the only input criterion on DEA model (a linear programming technique). Their study suggests that economic growth could significantly improve health care expenditure.

Using data for the period 2000-2015 on a panel data regression framework, Some et al. (2019) conducted an empirical study concerning the relationship between economic growth, medical industry, and healthcare industry in the case of 48 African countries. Their study found that health expenditure has a direct and important impact on economic growth, but also that total health expenditure promotes economic growth.

In another study, Rizvi (2019) used data from a sample of 20 South East Asian and Pacific developing countries from 1995 to 2017 to determine the effect of health expenditure on economic growth. For the attainment of this goal, the standard neoclassical Solow Growth Model at steady-state level was taken as theoretical framework and a production function was created adding institutional quality corresponding to government effectiveness alongside primary education, population increase, etc. The results of the study showed that if health expenditure adjusted to quality increased by 100%, then there will be an increase on growth by 5%.

Yang (2019) used a panel threshold model and panel data from 21 developing countries from 2000 to 2016 to analyze the relationship between economic growth and national expenditure for health sector on various levels of human capital. The results showed that the relationship between economic growth and health expenditure is related to human capital. Specifically, when human capital level is low, economic growth and medical expenditure are negatively correlated and are statistically significant. When human capital is on medium level, the impact of health expenditure on economic growth is positive but not statistically significant. When the level of human capital is high, the positive impact of health expenditure on economy is being enhanced considerably.

Esen and Kenili (2022) examined the consequences of health expenditure on economic growth in Turkey for the period 1975-2018.The variables used were household consumption, life expectancy, trade and foreign direct investments as control variables. For the long-run relationship among variables and short-run causality, the Johansen test and Granger test were used respectively. The results of the paper showed that there is a long-run relationship among variables and one-way causal relationship with direction from health expenditure to economic growth.

### CO_2_ Emissions and Health Expenditure

One of the most important environmental degradation indices is the increase of CO_2_ emissions. The increase of quantity of CO_2_ emissions negatively affects the residents’ health, leading to the outbreak of chronic diseases thus changing health expenditure. Researchers that have studied this fact, even though they have used different econometric methodologies, they concluded that the increase of CO_2_ emissions, increases health expenditure.

Dhrifi (2018) examines the consequences of environmental degradation of the quality of institutions and other macroeconomic variables on health using data from 45 African countries during the period 1995–2015. In order to proceed with this, the empirical analysis is conducting with GMM method for the problem solution of endogenous variables. The findings demonstrate that, on the one hand, there is a negative relationship between environmental degradation and health and on the other hand a positive relationship between quality’s institution and health. Moreover, direct and negative consequences of environmental degradation on health can be reduced from indirect and positive impact through quality’s institution and macroeconomic variables.

Taghizadeh-Hesary (2020) have applied the panel vector error correction model (VECM) and the panel generalized moment method (GMM) to analyze data from ten countries of South East Asia from 2000 to 2016. The study found that the increase of CO_2_ emissions which is caused by fossil fuels emissions, will cause an increase in per capita expenditures in health, while the use of renewable energy will decrease the per capita expenditures in health.

Gündüz (2020) used the hidden cointegration approach and crouching error correction model to analyze the impact of the US carbon footprint on health expenditure. The study used time series data from 1970 to 2016. The results showed that there is a long-run relationship between carbon footprints. Furthermore, the results showed that an increase on carbon footprint by 1% will increase health expenditure by 2.04%.

Oyelade et al. (2020) used the panel quantile regression to study the impact of CO_2_ emissions and public health expenditure on English-speaking countries of West Africa such as Gambia, Sierra Leone, Liberia, Ghana, and Nigeria from 1990 to 2013. The results of their study showed that the increase of CO_2_ emissions increase public health expenditure in all examined countries.

Bouchoucha (2021) examines the relationship between environmental degradation, health, and institutional quality on 17 countries of Middle East and North Africa (MENA). For the long-run relationship among variables, he used a panel cointegration analysis for the period 1996-2018 and the FMOLS and DOLS methods. The results of this study present that environmental degradation negatively affects the health situation on MENA countries in the long run. However, the effect of environmental degradation on health can be ameliorated through the presence of good institutional quality.

Akbar et al. (2021) used a panel VAR model to analyze the relationship between health expenditure, carbon dioxide emissions, and human development index (HDI) on 33 OECD countries from 2006 to 2016. For this purpose, an autoregression method based on the generalized method of moment estimations to test this relationship was used. The results showed that there is a bilateral causal relationship between health expenditure and carbon emissions, denoting that carbon dioxide emissions significantly increase health expenditure.

However, not all researchers reached the same conclusions regarding the relationship between CO_2_ emissions and healthcare expenditure. More specifically, Erdogan et al. (2020) have used a causality panel test developed by Konya in 2008 in order to analyze the relation between CO_2_ emissions and medical expenditures in BRICS (Brazil, Russia, India, China, South Africa) countries and Turkey from 2000 to 2016. The results show that only in the case of China, there was a unilateral positive relation between healthcare expenditure and CO_2_ emissions, with direction from CO_2_ emissions to health expenditure. On other selected countries, this relationship has not been identified as statistically significant.

The number of studies that detect the negative relationship between CO_2_ emissions and health expenditure are limited. Zaidi and Saidi (2018) investigated the relationship between health expenditure, CO_2_ emissions, and economic growth using data from Sub-Saharan African countries for the period 1990-2015. For the analysis of the relationship between variables the panel ARDL and VECM Granger causality were used. The results of their study featured that CO_2_ emissions have negative consequences on health expenditure. Specifically, the increase of CO_2_ emissions by 1% reduces health expenditure by 0.066%.

### CO_2_ Emissions and Economic Growth

Given that the model of economic growth for developing countries consumes a lot of energy, most researchers examine the relationship between carbon dioxide emissions and economic growth on developing countries. The different econometric models that study the relationship between carbon dioxide emissions and economic growth give two different conclusions: positive correlation and negative correlation.

When a country uses traditional energy, the researchers have realized that economic growth will increase CO_2_ emissions.

Adamu et al. (2020) used dynamic ordinary least squares (DOLS) and ARDL cointegration methods as tests to analyze the relationship between the transfer of CO_2_ emissions of Nigeria, the economic growth, and urbanization of rural population from 1971 to 2018. The results of this study showed that the immigration of rural population to the cities and economic growth led to a significant increase of CO_2_ emissions.

Öztürk and Suluk (2020) applied the Generalized Moment Method (GMM) and panel data from G7 countries from 1991 to 2014 in order to study the relations between CO_2_ emissions, consumption energy, and economic growth. In their study, they found a bidirectional causal relation between CO_2_ emissions and economic growth.

Ișik et al. (2020) used panel bootstrap cointegration in order to examine how the increase of renewable energy source consumption and international tourist revenue has affected CO_2_ emissions in the case of G7 countries from 1995 to 2015. They conclude that the increase of renewable energy source consumption affects the increase of tourism income in the case of France, Italy, and the UK. On the contrary, CO_2_ emissions in the case of the USA have a negative effect.

Adebayo and Akinsola (2021) analyzed time series data from 1971 to 2018 using the wavelet coherence method and Granger and Toda-Yamamoto causality techniques to investigate the causal relationship between energy consumption, CO_2_ emissions, and economic growth in Thailand. The results show a bilateral causal relationship between economic growth and carbon dioxide emissions. Moreover, both the short- and long-run CO_2_ emissions are positively correlated with GDP increase.

Kong (2021) used the asymmetric ARDL model and data from 1985 to 2019 in order to analyze the impact of economic growth, energy consumption, and foreign indirect investment on CO_2_ emissions in China. The results show that real GDP has significant positive impact on CO_2_ emissions.

Regmi and Rehman (2021) in order to reveal the impact of CO_2_ emissions on energy use, energy consumption, fossil fuels, increase population, and economic progress of Nepal used time series data from 1971 to 2019 and ARDL cointegration. The results, both in the long-run analysis and the short-run analysis, showed that energy consumption from fossil fuels have impact on CO_2_ emissions. Also, the results of Granger causality verify the one-way connection between variables.

Iheonu et al. (2021) used the STIRPAT (stochastic impacts by regression on population, affluence, and technology) to study the impact of economic growth, international trade, and population urbanization on CO_2_ emissions, on 34 countries of Sub-Saharan Africa for the period 1990–2016. The empirical findings reveal that GDP increases CO_2_ emissions in countries where the existing CO_2_ emissions level is low. The international trade improves environmental sustainability in countries where the existing levels of CO_2_ emissions are on a highest grade. The study also reveals a bilateral causal relationship between economic growth, international trade, urbanization, and CO_2_ emissions.

Ebrahimzadeh et al. (2021) studied the relationship between taxation, private sector investment, and other economic indices relating to economic growth, urbanization, and CO_2_ emissions for Iran using the Bayesian causal map (BCM) for the period 1980–2018. On their results, they realized that urbanization in Iran, different economic indices have different repercussions on CO_2_ emissions but all drive to the increase of CO_2_ emissions.

Li et al. (2021) used the STIRPAT (stochastic impacts by regression on population, affluence, and technology) model on panel data in 30 provinces for China from 2011 to 2017 to investigate the impact of energy structure and digital economy on carbon dioxide emissions. The results show that the increase of energy structure on non-resource energy provinces and mainly based on coal have larger impact on carbon dioxide emissions in relation to resource-based provinces. The energy structure based on coal has significant motivating effect on carbon dioxide emissions. With the development of digital economy, the impact of coal-based energy structure on carbon emissions is gradually decreasing. This effect is more significant in non-resource-based provinces and eastern China, but not significant in resource-based cities and central and western China.

Mongo et al. (2021) used the ARDL model for 15 European countries and data for the period 1991–2014 to analyse the impact of per capita GDP, the environmental innovation, the consumption of renewable energy resources, and economic openness of carbon dioxide emissions. The paper’s results indicated that, in the long run, environmental innovations tend to decrease CO_2_ emissions whereas in the short run, the result is adverse denoting the existence of recovery.

### Health Expenditure, CO_2_ Emissions, and Economic Growth

There is little research literature on the relationship between CO_2_ emissions, health spending, and economic growth. Wang et al. (2019) used data for Pakistan in the period from 1995 to 2017 and the autoregressive distributed lag (ARDL) model to study the dynamic relationship between carbon dioxide emissions, health expenditure, and economic growth under the condition of gross-fixed capital formation and per capita trade. The results of their work showed that there is a long-standing relationship between health expenditure, CO_2_ emissions, and economic development in Pakistan. The results also showed that there is a two-way causal relationship between health spending and carbon emissions, as well as health spending and economic growth.

Atuahene et al. (2020) used data from 1960 to 2019 and the generalized method of moments (GMM) model to study the relation between CO_2_ emissions, economic growth, and healthcare expenditure in the case of China and India. They conclude that there is a significant relation between the three variables. They also showed that CO_2_ emissions have a significant positive impact on healthcare expenditure in both countries, while economic development has a negative effect on healthcare expenditure.

In the case of more than 160 countries, Coccia (2021) studied whether each country’s gross domestic product (GDP) per capita, healthcare expenditure, and air pollution are the key factors related to COVID-19 mortality. The results of the paper suggest that countries with a low average COVID-19 mortality rate have high health spending >7.5% of GDP, as well as high health spending per capita >$2,300.

Li et al. (2022) use the Fourier autoregressive distributed lag (ARDL) model to study the correlation between health expenditure, CO_2_ emissions, and GDP fluctuations in the BRICS from 2000 to 2019. The results of their work showed that there is a long-standing relationship between the three variables only in Brazil and China. In India, there is a two-way causal relationship in CO_2_ emissions and health expenditure, while other countries have shown a one-way causal relationship between CO_2_ emissions and health expenditure, as well as between CO_2_ emissions and economic growth.

Ageli (2022) uses the Bootstrap Autoregressive Distributed Lag (BARDL) cointegration model to examine whether there is a short- and long-term relationship between per capita health expenditure, GDP per capita, and per capita CO_2_ emissions in the case of Saudi Arabia between 1995 and 2021. The results of the paper reject the integration relationship between the variables under examination. Empirical results of causality show a one-way causal relationship between GDP per capita and health expenditure, as well as between green energy and per capita CO_2_ emissions. The data also show a two-way relationship between health spending and CO_2_ emissions.

## Data

In order to study the relation between per capita healthcare expenditure, per capita GDP (proxy for economic growth) and per capita greenhouse emissions (proxy for environmental degradation) in the case for G7 countries, we have analyzed a panel model for the period between 2000 and 2018. The variable for health has been calculated based on the current per capita healthcare expenditure expressed in US dollars in purchasing power parity. The variable for environment represents the per capita greenhouse emissions (which generally represents CO_2_ emissions in the literature) measured in metric tonnes per capita. GDP per capita, as an index of economic growth level, has been calculated based on current prices expressed in US dollars in purchasing power parity.

The study sample includes the team of G7 most developed, most wealthy, and most liberal economies according to IMF. This team consists of the following countries; Canada (CAN), Germany (DEU), France (FRA), the United Kingdom (GBR), Italy (ITA), Japan (JPN), and the United States (USA). The period under investigation is 2000 to 2018. Stata 14.0 και Eviews 12.0 have been used for the economic analysis of the paper. All variables, with their description as well as the data sources, are presented at Table 2.

**Table 2:** Data description

Variables	Description	Source
HEC	Current health expenditure per capita (PPP current international $) last update: 16/12/2021	World Health Organization Global Health Expenditure database (apps.who.int/nha/database)
GDPC	GDP per capita, PPP (current international $) last update: 16/12/2021	International Comparison Program, World Bank | World Development Indicators database, World Bank |
GHGC	Greenhouse gas emissions per capita (measured in metric tones per capita) (online data code: T2020-RD300 ) last update: 18/08/2021	Source of data: Global Carbon Project. (2021). 10.18160/gcp-2021

**Fig. 3:**
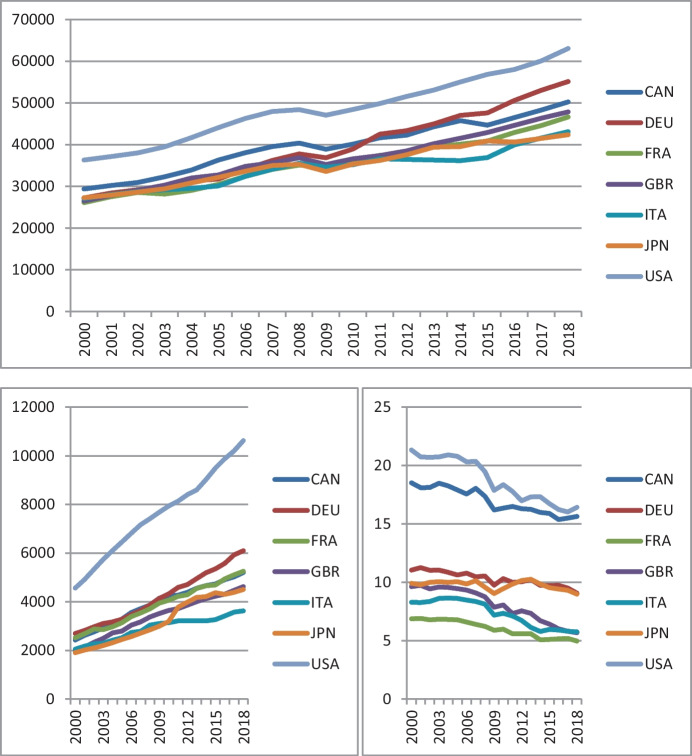
GDP, health expenditure, and CO_2_ emissions (per capita)

Figure 3 presents GDP per capita, PPP (current international $), health expenditure per capita, PPP (current international $), and CO_2_ emissions (per capita)

According to Fig. 3, the USA depicts the highest GDP and health expenditures, while Italy has the lowest GDP and health expenditures. Also, the USA has the highest CO_2_ emissions per capita while France has the lowest CO_2_ emissions per capita. Table 3 presents in detail all descriptive statistics of all the G7 countries under investigation.

**Table 3:** Description of GDP, health expenditure, and CO_2_ emissions (per capita) statistics

Economic variables	Canada			Germany			France			United Kingdom		
	HEC	GDPC	GHGC	HEC	GDPC	GHGC	HEC	GDPC	GHGC	HEC	GDPC	GHGC
Mean	3914	39677	16.96	4204	39265	10.30	3862	35357	6.016	3394	36689	8.071
Maxi	5199	50239	18.52	6098	55142	11.25	5250	46620	6.910	4619	47863	9.740
Mini	2429	29362	15.38	2691	27210	9.07	2516	26101	4.960	1927	26483	5.680
Sd. Dev	864	6238	1.111	1093	8747	0.616	870	6101	0.720	822	6174	1.446
Skew	−0.25	−0.147	0.068	0.253	0.291	−0.188	0.009	0.162	−0.106	−0.25	0.139	–0.342
Kurt	1.812	2.010	1.419	1.774	1.877	2.02	1.712	1.974	1.454	1.887	2.154	1.649
J-B	1.327	0.844	1.991	1.392	1.267	0.864	1.313	0.916	1.926	1.181	0.627	1.816

Economic variables	Italy			Japan			United States		
	2907.7	34298	7.34	HEC	GDPC	GHGC	HEC	GDPC	GHGC
Mean	3624.0	43119	8.65	3211	35117	9.74	7604	48565	18.76
Maxi	2049.4	27084	5.75	4503	42386	10.25	10623	63064	21.33
Mini	490.6	4603	1.149	1908	27282	8.99	4564	36334	16.02
Sd. Dev	–0.359	0.113	–0.258	944	4844.6	0.370	1797	7900	1.899
Skew	1.838	2.182	1.398	0.083	–0.118	–0.676	–0.029	0.085	–0.034
Kurt	1.475	0.570	2.243	1.400	1.805	2.431	2.008	2.079	1.360
J-B	2907.7	34298	7.34	2.048	1.173	1.705	0.781	0.693	2.132

Source: Author’s calculations

The mean GDP per capita varies from 34,298 (for Italy) to 48,565 (for the USA) (PPP current international $). The mean for per capita healthcare expenditure varied from 2907.7 (for Italy) to 7604 (for the USA) (PPP current international $). The mean for per capita greenhouse emissions varied from 6.016 to 18.76 (metric tonnes) for France and the USA respectively. In the majority of the countries, variables are left-skewed, and all variables have low-spread peaks (< 3). Finally, all analysis results seem to follow a normal distribution based on Jarque-Bera test and there are fewer outliers which meets the requirements of the empirical analysis.

## Methodology

The model used for the purpose of the current study with the logarithmic form for the variables is the following:

1 $${LHEC}_{it}={\alpha}_0+{\beta}_1{LGDPC}_{it}+{\beta}_2{LGHGC}_{it}+{u}_{it}$$

where *t*=1,…,T and *i*=1,…,*N* index the time-series and cross-sectional units, respectively. LHEC in the natural logarithm of health expenditure per capita, LGDPC is the natural logarithm of GDP per capita, LGHGC is the natural logarithm of greenhouse gas emissions per capita, and *u*_*it*_ is the error term including all unobserved factors.

In the model above, *β*_1_ is the coefficient measuring the impact of per capita GDP on per capita healthcare expenditure. The greater the per capita GDP, the greater the per capita healthcare expenditure. Therefore, the coefficient is expected to take a positive sign. Nevertheless, the magnitude of the coefficient determines whether the per capita healthcare expenditure is a basic or a luxury product. More specifically, macroeconomic theory suggests the following (Abdullah et al. 2017).

If 0 ≤ *β*_1_ ≤ 1, then per capita healthcare expenditure is a basic product.

If *β*_1_ > 1, then per capita healthcare expenditure is a luxury product.

### Random Effects Vs. Fixed Effects Estimation

Econometric modeling of panel data usually applied two basic approaches, the one of fixed and the one of random effects. In order to investigate these two approaches, Hausman (1978) test is applied. The test is based on the idea that the amount of coefficient estimation derived from the estimation of fixed effects should not differ systematically from the estimation of random effects.

### Cross-Sectional Dependence

In order to use panel unit root tests, we should examine if there is a cross -sectional dependency on panel data. If there is no cross-sectional dependency, then we could use the first- generation panel unit root test (Levin et al. (2002), Breitung (2000), Im et al. (2003), Hadri (2000), Maddala and Wu (1999), and Choi (2001). If there is a cross-sectional dependency in the data, then first-generation panel unit root test cannot be applied. In the latter case, second-generation panel unit root tests can be used (SURADF, CADF, and CIPS) which take into account cross-sectional dependency. Cross-sectional dependency can be explained in econometric terms as the integration of residuals between the model units of panel data given in Equation (1). From an economic point of view, it could be explained that in a state where units which form the panel data are affected by a shock, then all unit in the table are affected.

### Slope Homogeneity Tests

When analysing panel data, it is considered that the non-observed heterogeneity is captured from individual-specific constants, which are regarded as fixed or random. Ignoring such form of heterogeneity could lead to biased results. Therefore, it is important to test the hypothesis of homogeneity slope before applying the standard techniques of panel data.

This hypothesis, in other words the coefficient heterogeneity, is tested using the «Delta Test» presented by Pesaran and Yamagata (2008) as an alternative to Swamy (1970) slope homogeneity. Delta test statistic and the adjusted bias version of mean and standard deviation of Delta statistics can be calculated as follows:

2 $$\tilde{\Delta}=\sqrt{N}\left(\frac{N^{-1}\tilde{S}-k}{\sqrt{2k}}\right)$$

where

*N* is the number of cross-section units, *k* is the independent variables, and $\tilde{S}$ represents the Swamy (1970) statistic.

3 $$\tilde{S}=\sum \limits_{i=1}^N{\left({\hat{\beta}}_i-{\hat{\beta}}_{WFE}\right)}^{\prime}\frac{{\overline{x}}^{\prime}\overline{x}}{{\hat{\sigma}}_i^2}\left({\hat{\beta}}_i-{\hat{\beta}}_{WFE}\right)$$

where ${\hat{\beta}}_i$ are the heterogenous coefficients for each cross section, ${\hat{\beta}}_{WFE}$ shows the weighted coefficient of fixed estimators (weights are constructed using ${\hat{\sigma}}_i^2$), $\overline{x}$ is the matrix of the deviations from the mean of explanatory variables.

In the case of normally distributed error terms, the bias adjustment of the mean variance $\tilde{\Delta}$ can be expressed as:

4 $${\tilde{\Delta}}_{adj}=\sqrt{N}\left(\frac{N^{-1}\tilde{S}-E\left({\tilde{z}}_{iT}\right)}{\sqrt{Var\left({\tilde{z}}_{iT}\right)}}\right)$$

where $$E\left({\tilde{z}}_{iT}\right)=k$$ and $$Var\left({\tilde{z}}_{iT}\right)=\frac{zk\left(T-k-1\right)}{T+1}$$

### Panel Unit Root Tests

In the literature review of panel data, series stationarity tests can be categorised in two groups. The first-generation panel unit root tests which are considered insufficient due to the fact that the fail to take into consideration the cross-sectional dependency between panel units produce bias results (Baltagi and Pesaran, 2007). The second-generation panel unit root test, on the other hand, allows the cross-sectional dependence on panel units. If the hypothesis of cross-sectional dependence and slope homogeneity of the data is rejected, we could use the second generation unit root Covariate-Augmented Dickey-Fuller (CADF) test by Pesaran (2007) Cross-Sectionally Im-Pesaran-Shin (CIPS). CADF unit root test can be calculated using the following equation:

5 $$\Delta {y}_{it}={\alpha}_i+{g}_it+{b}_i{y}_{i,t-1}+{c}_i{y}_{t-1}+\sum \limits_{j=0}^p{d}_{ij}\Delta {y}_{t-j}+\sum \limits_{j=0}^p{\delta}_{ij}\Delta {y}_{i,t-j}+{e}_{it}$$

where *i* = 1, …, *t* , *α*_*i*_ is the constant term, *t* represents the trend, *y*_*i*, *t* − *j*_ represents the time lag, and *e*_*it*_ represents the error term.

In order to analyze the results of the CADF test for the whole panel, a Cross-Sectionally Im-Pesaran-Shin (CIPS) test could be applied as below:

6 $$CIPS\left(N,T\right)={N}^{-1}\sum \limits_{i=1}^N{CADF}_i$$

The null hypothesis of Covariate-Augmented Dickey-Fuller (CADF) test denotes the existence of unit root in the series.

### Panel Cointegration Tests

If the unit root test is rejected, we could use the Westerlund (2007) cointegration test. Westerlund (2007) calculates four different statistics based on the error correction model between the unit panel and the whole panel. *G*_*t*_ and *G*_*a*_ indicate cointegration relation for at least one cross-sectional units, while *P*_*t*_ and *P*_*a*_ show this relation for the whole panel (Persyn and Westerlund, 2008). Even though the Westerlund (2007) panel cointegration test allows heterogeneity and cross-sectional dependence across panel units, it assumed that all variables used in the panel are first-order integrated.

### Augmented Mean Group (AMG) Estimator

In the case where a cross-sectional dependency exists and variable are cointegrated, we move on to estimate the model using the augmented mean group (AMG) estimator by Eberhardt and Teal (2010). The AMG panel estimator as adopted by Eberhardt (2012) for cross-section *i* = 1, …, *N* and time period *t* = 1, …, *T* is estimated from the following model:

7 $${y}_{it}={\beta}_i{x}_{it}+{u}_{it}\;\textrm{for}\;i=1,\dots, N\kern0.37em \textrm{and}\;t=1,\dots, T$$

8 $${u}_{it}={\alpha}_{1i}+{\lambda}_i{f}_t+{\varepsilon}_{it}$$

9 $${x}_{it}={\alpha}_{2i}+{\lambda}_i{f}_t+{\gamma}_i{g}_t+{e}_{it}$$

where *y*_*it*_ and *x*_*it*_ are the observed series , *β*_*i*_ is the slope of the specific country on the observed regressor, *u*_*it*_ is the sum of the non-observed common factors and *ε*_*it*_ are the error terms.

### Causality Test

It should be noted that Baloch Mahmood and Zhang (2019) argue that Granger’s causality approach fails to account for heterogeneity and cross-layer dependence in panel data. This paper therefore extends this analysis by presenting the results of the Granger causality test using the Dumitrescu and Hurlin causality technique (2012). The null hypothesis assumes that there is no homogeneous Granger causality, and the alternative supports the existence of at least one causal relationship in the data set under study. The null hypothesis posits that there is no homogenous Granger causality, and the alternative supports the existence of at least one causal association in the studied data set.

The regression suggested by Dumitrescu and Hurlin (2012) for detected causality on panel data is as follows:

10 $${y}_{i,t}={\alpha}_i+\sum \limits_{k=1}^K{\gamma}_{ik}{y}_{i,t-k}+\sum \limits_{k=1}^K{\beta}_{ik}{x}_{i,t-k}+{e}_{i,t}\kern0.37em \textrm{with}\;i=1,\dots, N\;\textrm{and}\;t=1,\dots, T$$

where *y*_*i*, *t*_ and *x*_*i*, *t*_ are the observations two stationary variable for individual *i* in period *t*. The coefficients are allowed to differ between individuals but there are regarded as unchanged over time. The order of lag *K* is the same for all individuals and the panel should be balanced.

Dumitrescu and Hurlin test assumes that there could be causality for some individuals, but not necessarily for all. Therefore, the alternative hypothesis is:

$$H_1:\beta_{i1}=\dots=\beta_{ik}=0\;\forall i=1,\dots,N_1.$$

$$H_1:\beta_{i1}\neq0\;or\dots or\;\beta_{ik}\neq0\;\forall i=N_1+1,N_1+2,\dots,N.$$

## Empirical Analysis

When analysing panel data, Hausman (1978) test enables us to choose between a fixed effects models and a random effects model. The null hypothesis states that the preferred model is the one of random effects, whereas the alternative hypothesis states as preferred the fixed effected model. Table 4 presents the Hausman (1978) test results.

**Table 4:** Hausman test

Test summary	Chi-Sq. statistic	Chi-Sq. d.f.	Prob.
Cross-section random	20.892060	2	0.0000

Source: Author’s calculations

The results from Table 4 reject the hull hypothesis, so we could say that the fixed effects model is the best suited one. In order to test for cross-sectional dependency among the residuals, we use the tests by Breusch-Pagan LM (1980), Pesaran-scaled LM (2004), Pesaran CD (2004), and Baltagi Feng and Kao (2011) bias-corrected scaled LM. The null hypothesis indicates that there are no relations among cross sections.

11 $$Ho:{\hat{\rho}}_{ij}= Corr\left({u}_{it},{u}_{jt}\right)=0\; for\;i\ne j$$

Results from the above tests are presented in Table 5.

**Table 5:** Cross-sectional dependence and homogeneity test results

Test	Statistic	*p-*value
Cross-sectional dependence test (H_0_: no cross-sectional dependence)		
Breusch-Pagan LM	124.9231	0.000
Pesaran-scaled LM_s_	16.03568	0.000
Bias-corrected scaled LM_p_	15.84124	0.000
Pesaran CD_BC_	8.128078	0.000
Homogeneity test (H_0_: slope coefficients are homogeneous)		
$\tilde{\Delta}$	38.696	0.000
${\tilde{\Delta}}_{adj}$	39.743	0.000

$\tilde{\Delta}$ and ${\tilde{\Delta}}_{adj}$ test denote the slope homogeneity tests proposed by Pesaran and Yamagata (2008)

Source: Author’s calculations

Results of all tests presented in Table 5 suggest that we could reject null hypothesis of cross-sectional dependence in 1% significance level. Also, at the same table, the estimated statistical tests and the corresponding *p-*values suggest that the null hypothesis of homogenous slopes should be rejected, and they suggest that it is important to consider the slope heterogeneity.

Therefore, we could say that first-generation unit root tests possibly produce ineffective results. So, we use second-generation unit root tests by Pesaran (2007) CIPS which take into consideration the cross-sectional dependence and heterogeneity.

As suggested by Table 6, the null hypothesis of unit root is rejected in the first differences. Therefore, all variables, are first-order integrated *Ι*(1). Following the existence of variable stationarity, we use the stationarity test by Westerlund (2007) which allows the heterogeneity and cross-sectional dependency among panel units and assumes that all variables used at panel data are integrated at first-order.

**Table 6:** Pesaran CADF panel unit root test

	Pesaran-CIPS			
	Intercept		Intercept and trend	
Variable	*t*-stat	Prob.	*t*-stat	Prob.
LHEC	−1.625	>0.10	−1.561	>0.10
LGDPC	−2.173	>0.10	−2.280	>0.10
LGHGC	−1.934	>0.10	−3.301*	<0.01
ΔLHEC	−2.326***	<0.10	−2.854***	<0.10
ΔLGDPC	−2.224***	<0.10	−2.762***	<0.10
ΔLGHGC	−2.788*	<0.01	−2.780*	<0.01

Critical values: −2.36, −2.18, −2.08 (intercept), and −2.88, −2.70, −2.60 (intercept and trend) * and ** indicate 1% and 5% level of significance respectively, Δ is first difference; the lag lengths from cross-sections were selected using Modified Akaike Information Criterion (MAIC)

Source: Author’s calculations

Tests shown in Table 7 reject the null hypothesis of no-cointegration of cross-sectional units and whole panel, which implies that there are fixed and long-term relations among these variables. The results of long-term coefficients of cointegration using the augmented mean group (AMG) method are presented in Table 8.

**Table 7:** Error correction panel cointegration test results

Statistic	Value of the test	*Z*-value	*p-*value
*G*_t_	−2.786	−3.127	0.017**
*G*_a_	−8.214	−5.174	0.049**
*P*_t_	−9.101	−3.043	0.006*
*P*_*a*_	−10.221	−5.735	0.001*

* and ** indicate 1% and 5% level of significance respectively,

**Table 8:** Long-run cointegrating coefficients

Country	LGDPC	LGHGC
Canada (CAN)	1.039*	−0.972*
Germany (DEU)	0.968*	−0.817*
France (FRA)	0.887*	−0.584*
United Kingdom (GBR)	0.855*	−0.420*
Italy (ITA)	0.815*	−0.281*
Japan (JPN)	1.491*	−3.325*
United States (USA)	1.017*	−0.702*

*, **, and *** are respectively significant at 1%, 5%, and 10%.

As presented in Table 8, elasticities of greenhouse gas emissions per capita which are derived from the AMG estimator, are all negative and statistically significant for all countries in G7 group. The country with the highest elasticity is Japan. On the contrary, GDP per capita elasticities derived from the same estimator are all positive and statistically significant. As mentioned in Equation (1) if 0 ≤ *β*_1_ ≤ 1, then the per capital health care expenditure is a basic product. Therefore, this is the case of Germany, France, the United Kingdom, and Italy. If *β*_1_ > 1, then the per capital health care expenditure is a luxury product, which is the case of Canada, Japan, and the USA. Results of the short-term causality tests for every country are presented in Table 9, where the direction of causal effect is noted with (⇒) or (⇐) for unidirectional causal relations.

**Table 9:** Results of causality test

Country	ΔLGDPC,ΔLHEC	ΔLHEC,ΔLGHGC	ΔLGHGC,ΔLGDPC
Canada (CAN)	≠	⇐**	≠
Germany (DEU)	≠	≠	≠
France (FRA)	≠	≠	≠
United Kingdom(GBR)	≠	≠	≠
Italy (ITA)	≠	⇐***	≠
Japan (JPN)	⇒**	⇐**	≠
United States (USA)	≠	≠	≠

*, **, and *** indicate the rejection of the null hypothesis at 1%, 5%, and 10% significance level.

The results of the above table show a unidirectional causal effect from GDP per capita towards health expenditure per capita for the case of Japan, and unidirectional causal effect from the greenhouse gas emissions per capita towards health expenditure per capita for the case of Canada, Italy, and Japan. For Germany, France, United Kingdom, and the USA the results show no causal relations.

The causality between health expenditure per capita, GDP per capita, and greenhouse gas emissions per capita for all G7 countries, has been analyzed through the Dumitrescu and Hurlin (2012) causality test, and its findings are presented in Table 10. Dumitrescu and Hurlin (2012) causality test is being calculated based on three different statistic values.

**Table 10:** Dumitrescu and Hurlin (2012) test results

Null hypothesis	W-Stat.	Zbar-Stat.	Prob.
DLGDPC does not homogeneously cause DLHEC	2.50224	0.04990	0.9602
DLHEC does not homogeneously cause DLGDPC	1.57963	-0.74669	0.4552
DLGHGC does not homogeneously cause DLHEC	4.76435	2.00305	0.0452
DLHEC does not homogeneously cause DLGHGC	3.06484	0.53566	0.5922
DLGHGCdoes not homogeneously causeDLGDPC	1.32080	-0.97018	0.3320
DLGDPCdoes not homogeneously cause DLGHGC	1.74089	-0.60746	0.5435

Dumitrescu and Hurlin (2012) causality test, revealed a unilateral causality from the greenhouse gas emissions per capita towards health expenditure per capita.

## Discussion

The main purpose of this work is to investigate the environmental impact and development on health expenditure. The empirical analysis is based on the G7 countries over the period 2000-2018. From the brief statistical analysis of labor variables we observe that the average US GDP per capita is 48565 (PPP current international $) which makes it the highest-income economy among the G7 countries, followed by Canada and Germany with 39677 and 39265 GDP per capita respectively, while Italy with 34298 is the economy with the lowest GDP per capita. In terms of average greenhouse gas emissions per capita, the USA with 18.76 (metric tonnes) has the highest average per capita greenhouse emissions, followed by Canada with 16.96 per capita greenhouse gas emissions, while France with 6,016 has the lowest per capita greenhouse emissions. Also, average health spending per capita is with 7604(PPP current international$) highest in the USA, followed by Germany with 4204, while Italy with 2907.7 has the lowest.

Later on, the analysis of the panel data with Hausman’s control (1978) enabled us to choose the fixed effects model as the most appropriate. In addition, the findings of the four dependency tests rejected the zero hypothesis of cross-sectional independence, as well as slope homogeneity. Pesaran (2007) CIPS’s second-generation unit root test that takes into account cross-sectional dependence and heterogeneity showed that all variables are integrated first-order I(1). Therefore, we proceeded to test the integration of Westerlund (2007) allowing heterogeneity and cross-sectional dependence. The results show that there is a stable and long-term relationship between per capita health expenditure, GDP per capita, and per capita CO_2_ emissions. The results of the cointegration are in line with the work of Khoshnevis and Khanalizaden (2017), in the MENA countries, Bilgili et al. (2021) for Asian countries, as well as Ganda (2021) for the BRICS countries where variables move together in the long term. With these results, the research proceeds by presenting the long-term relationships with the augmented mean group (AMG) method and answering the first research questions and hypotheses raised in the introduction of the paper. The results obtained by the AMG estimator show that average health expenditure per capita is negatively related to average greenhouse gas emissions per capita in all G7 countries. The discrepancy in these results can be explained by the fact that different studies used different variables to represent environmental pollution. In most studies, as well as the current one, we include only CO_2_ emissions in per capita greenhouse gas emissions. Specifically, an increase in per capita greenhouse gas emissions by 1% in Japan will reduce per capita health spending by 3.32%; for Canada, the reduction in head health expenditure will be 0.97%, while for Italy, the reduction will be 0.28%. The differences between countries represent the degree of industrialization of each country. These findings contrast with the results of Zaman and Abd-el Moemen’s (2017) work on the 14 Latin American economies, while agreeing with Zaidi and Saidi’s work (2018) for sub-Saharan African countries, Ganda's work (2021) for the BRICS countries, and Bayar et al.’s work (2021) for EU countries. Moreover, the findings show that the growth of average GDP per capita is positively correlated with average greenhouse gas emissions per capita in all G7 countries. Specifically, an increase in average GDP per capita by 1% will increase average per capita greenhouse gas emissions in Japan by 1.49%, in Canada by 1.03%, in the USA by 1.01%, and in Italy by 0.81%. Our work confirms the results from the revised literature i.e., one of the most determinants of health expenditure is GDP per capita (see Gerdtham et al. (1998), Hitiris (1999), Erçelik (2018), Zaidi and Saidi (2018), and Bayar et al. (2021).

In addition, our results show that GDP per capita has a significant positive impact on health spending in all G7 countries, which can be explained by the fact that we conducted a study that included developed countries which are characterized by high standards of living that increase people's longevity and consequently reduce mortality risks. Further changes and technological developments in the medical field are expected to prolong people’s lives but also increase health-related costs.

Short-term causality with the Granger test for each G7 country answers the following research questions and hypotheses mentioned in the introduction and argues that in Canada there is a one-way causal relationship between per capita CO_2_ emissions and per capita health expenditure directed from per capita CO_2_ emissions to per capita health expenditure in Canadian countries, Italy and Japan, as well as a one-way causal relationship between health expenditure per capita and GDP per capita in the direction of GDP per capita to per capita expenditure for Japan. In other words, we would say that CO_2_ emissions and GDP per capita have a significant impact on health expenditure for these countries. The countries of Germany, France, and the United Kingdom did not show a statistically significant causal relationship. Finally, the findings of Dumitrescu and Hurlin (2012), for the G7 countries as a whole, revealed a unilateral causal relationship from greenhouse gas emissions per capita to health expenditure per capita. Therefore, we can say that in all G7 countries, CO_2_ emissions have a significant impact on health expenditure. A one-way causal relationship between CO_2_ emissions and healthcare costs was validated through various studies such as Chaabouni and Saidi (2017) and Erdogan et al. (2020) while other studies identified bidirectional causality such as Ullah et al. (2019) or no significant causality such as Erdogan et al. (2020).

## Conclusions

Health is one of the most important factors that decide the quality of human capital. Identifying the main and determining factors in healthcare spending is crucial for both researchers and policymakers. There are many factors that can affect the health status of the population, such as environmental health, socio-economic status, economic development, and environmental quality. As the quality of the environment deteriorates, the degradation of global environmental quality is a serious challenge to healthy living. Many papers have examined the relationship of health expenditure with different socio-economic indicators, such as income, inflation, globalisation, life expectancy, and the level of industrialization taking into account the needs of the future generation for the environmental dimensions they need to address.

The topic selected in this study addresses several topics of interest as to the determinants of healthcare costs such as environmental issues measured through greenhouse gas emissions, and actual GDP per capita that affect healthcare spending. Climate change has become an undeniable fact that poses a serious threat to the sustainable development of human survival, society, the economy, and the environment. There is an important link between high concentrations of greenhouse gases and climate change that there is the impact of climate change on public health. The main purpose of this work is to investigate the environmental impact on health expenditure of the G7 group of states in the period 2000–2018. In our work, we have included the G7 countries as the most developed countries on the planet, characterized by high living standards that increase the longevity of people and reduce mortality risks.

Economic growth, environmental degradation, and healthcare expenditure relations vary among all G7 countries. As a result, the negative external effects of low environmental quality due to impact on healthcare expenditures are unknown to the developed countries. Increasing healthcare expenditures is regarded as a major concern to all G7 governments and the understanding of their determinant factors could aid policy makers into developing suitable policies.

In conclusion, our work reveals the strong links between per capita CO_2_ emissions of GDP per capita and per capita health expenditure, as we consider these three elements as the key factors in determining health expenditure in the G7 countries to protect humans. Protecting human health is a necessity, and the global health crisis caused in recent years by the spread of COVID-19 has proven that no country is fully prepared to adequately handle a pandemic. Exploring the factors that can improve the health sector is vital and can help the state and local public health authorities find solutions to make this sector more resilient. Renewable energy sources or mild forms of energy or new energy sources or green energy are forms of exploitable energy derived from various natural processes, such as wind, geothermal, water circulation, and others. Therefore, it is a real challenge for any country to achieve sustainable economic growth which in turn will encourage governments to increase health spending.

With regard to the limitations of our work, we must stress out that our analysis focused on limited data collected on the G7 states. Therefore, future studies should expand the sample size. In addition, future research should look at countries at different levels of development and add other determinants of environmental or socio-economic variables that affect health expenditure.
